# Secreted Metabolites of *Bifidobacterium infantis* and *Lactobacillus acidophilus* Protect Immature Human Enterocytes from IL-1β-Induced Inflammation: A Transcription Profiling Analysis

**DOI:** 10.1371/journal.pone.0124549

**Published:** 2015-04-23

**Authors:** Shuangshuang Guo, Yuming Guo, Ayla Ergun, Lei Lu, W. Allan Walker, Kriston Ganguli

**Affiliations:** 1 State Key Laboratory of Animal Nutrition, College of Animal Science and Technology, China Agricultural University, Beijing, China; 2 Mucosal Immunology and Biology Research Center, Massachusetts General Hospital for Children and Harvard Medical School, Charlestown, Massachusetts, United States of America; 3 Department of Molecular Biology, Massachusetts General Hospital, Boston, Massachusetts, United States of America; 4 Section of Neonatology, Department of Pediatrics, The University of Chicago, Chicago, Illinois, United States of America; University of Florida, UNITED STATES

## Abstract

Combination regimens of *Bifidobacterium infantis * and *Lactobacillus acidophilus* have been demonstrated to prevent necrotizing enterocolitis (NEC) in clinical trials. However, the molecular mechanisms responsible for this protective effect are not well understood. Additionally, conditioned media from individual cultures of these two probiotics show strain specific modulation of inflammation using *in vitro* human intestinal NEC models. Here we report a transcription profiling analysis of gene expression in immature human fetal intestinal epithelial cells (H4 cells) pretreated with conditioned media from *B*. *infantis* (BCM) or *L*. *acidophilus* (LCM) prior to IL-1β stimulation. Compared with control media, the two probiotic-conditioned media (PCM) treatments altered the expression of hundreds of genes involved in the immune response, apoptosis and cell survival, cell adhesion, the cell cycle, development and angiogenesis. In IL-1β-stimulated cells, PCM treatment decreased the upregulation of genes in the NF-κB activation pathway and downregulated genes associated with extracellular matrix (ECM) remodeling. Compared with LCM, BCM showed more significant modulatory effects on ECM remodeling, reflected by a lower p value. IL-6 and IL-8 production was significantly reduced in IL-1β-stimulated cells pretreated with PCM (p<0.05), which was consistent with their altered gene expression. Western blot analysis showed that compared with IL-1β stimulation alone, PCM treatment attenuated the decrease of cytoplasmic IκBα and NF-κB p65 levels as well as the increase of nuclear NF-κB p65 levels in the stimulated cells (p<0.05). In conclusion, PCM treatment exerted anti-inflammatory effects in immature human fetal enterocytes primarily by modulating genes in the NF-κB signaling and ECM remodeling pathways. Additionally, some components of these signaling pathways, particularly the ECM remodeling pathway, were more profoundly affected by BCM than LCM.

## Introduction

As the incidence of premature birth increases and occurs at earlier gestational ages, the ill-equipped gastrointestinal tract is increasingly required to participate in the process of bacterial colonization within the extrauterine environment. The interaction between the immature intestinal tract and colonizing bacteria may lead to the most common gastrointestinal emergency of premature infants, necrotizing enterocolitis (NEC) [1]. This devastating condition is manifested by extensive intestinal inflammation of the distal intestine and affects almost 10% of all infants with a birth weight less than 1500 g [2,3]. Mortality associated with this life-threatening disease is as high as 40% to 50% in infants who require surgical intervention. Complications of the disease include short bowel syndrome, poor neurodevelopmental outcomes and growth restriction [4–6]. The most important risk factor for NEC is prematurity [7]. Accordingly, using established intestinal models for human gut development, this laboratory has demonstrated an immature expression of innate immune response genes resulting in exaggerated inflammatory responses to initial bacterial colonization, likely contributing to the development of NEC [1,8–10]. Therefore, a major strategy for preventing NEC is to find a means of reducing the immature inflammatory response and to accelerate the maturation of intestinal defenses [8].

Probiotics are defined as “live microorganisms which when administered in adequate amounts confer a health benefit to the host” [11]. *Bifidobacteria* and *Lactobacilli* are the predominant early colonizing organisms in the gastrointestinal tract of healthy breast-fed infants [12], but less prevalent in formula-fed or premature infants, who have the highest risk of NEC [13,14]. In clinical studies, the combined administration of *Bifidobacteria infantis* and *Lactobacillus aciodophilus*, given with expressed breast milk, significantly reduced the incidence and severity of NEC in very low birth weight (VLBW) infants [15,16]. Although effective in clinical trials, the mechanism responsible for the protective effect of probiotics in premature newborns is incompletely understood. In animal models of NEC, oral administration of *B*. *bifidum*, *B*. *infantis*, *L*. *rhamnosus* GG or *L*. *reuteri* showed beneficial effects via several mechanisms, including suppressed inflammation, improved intestinal barrier integrity or reduced apoptosis [17–21].

Although the protective effects of probiotics in preventing NEC are very promising in human and animal studies, some researchers remain appropriately concerned that the ingestion of intact bacteria by ill or immunocompromised preterm infants may induce a risk of infection and sepsis [22,23]. In addition, the use of bacteria in premature infants is not sanctioned by the US Food and Drug Administration. Thus, studies recently have been focusing on the protective effects of probiotic-conditioned media (PCM), the cultivation broth of probiotics subsequently filtered to remove all bacteria. The previous observations from our laboratory showed that PCM from co-cultured *B*. *infantis* or *L*. *acidophilus* decreased toll-like receptor (TLR) 2- and TLR4-dependent induction of proinflammtory cytokines by promoting the maturation of innate immune response gene expression in human fetal intestinal explants [8]. We also previously found that compared with conditioned media from *L*. *acidophilus*, media from *B*. *infantis* was more effective in reducing TNFα-induced IL-8 secretion in fetal enterocytes. We further demonstrated that *B*. *infantis*-conditioned media prevented the decrease of body weight, attenuated enterocyte apoptotic cell death, mitigated reduced mucin production and maintained ileal integrity in *Cronobacter sakazakii*-induced intestinal inflammation of newborn mice [24].

Microarray analysis allows mRNA expression to be assessed on a global scale and allows parallel assessment of gene expression for hundreds of thousands of genes relevant to intestinal structure, function and development. This feature makes it the most widely used method for profiling mRNA expression [25]. In our study, immature human intestinal epithelial cells were stimulated with IL-1β to induce an inflammatory state similar to NEC. Using microarray analysis, we investigated the expression of a large number of genes to help illustrate the mechanism by which PCM modulates the inflammatory response of the immature intestine, as seen in NEC. Given the previously shown strain specific anti-inflammatory effects of probiotics, we elected to investigate these effects using conditioned media from individually cultivated *B*.*infantis* and *L*.*acidophilus*.

## Materials and Methods

### Materials

Cell culture media, Dulbecco’s modified Eagles medium (DMEM), as well as nonessential amino acid (100×), glutamine (100×), antibiotic/antimycotic solution (100×), HEPES buffer (1 M) and sodium pyruvate (100 mM) were obtained from Gibco (San Diego, CA). Fetal bovine serum (FBS) was obtained from Atlanta Biologicals (Lawrenceville, GA). Humulin R regular insulin human injection (100 units/ml) was obtained from Lilly (Indianapolis, IN). Tissue culture plastics were obtained from Fisher Scientific (Pittsburgh, PA). The RNeasy Mini kits were obtained from Qiagen (Valencia, CA). SuperScript III first-strand synthesis supermix and SYBR GreenER qPCR SuperMix Universal were obtained from Life Technologies (Grand Island, NY). Recombinant IL-1β and enzyme-linked immunosorbent assay (ELISA) kits for IL-6 and IL-8 were obtained from R&D Systems (Minneapolis, MN). The nuclear and cytoplasmic extraction kit, protein assay reagents and ECL reagents were obtained from Thermo Scientific (Rockford, IL). Primary and secondary antibodies were obtained from Cell Signaling Technology (West Grove, PA), with the exception of primary antibody to histone H3, which was obtained from Abcam (Cambridge, MA). The Goat anti-rabbit IgG FITC conjugate was obtained from Zymed Laboratories Inc (San Francisco, CA). All other chemicals, unless specified, were purchased from Sigma-Aldrich (St. Louis, MO).

### Bacterial cultures and preparation of probiotic-conditioned media

The methods of probiotic cultivation and methods of isolating conditioned media used in the current experiments were previously described by Ganguli et al [8]. Briefly, *Bifidobaterium infantis* (ATCC No. 15697) and *Lactobacillus acidophilus* (ATCC No. 53103) were obtained from American Type Culture Collection (ATCC, Manassas, VA), cultured as recommended by ATCC, and stored individually in Mann-Rogosa-Sharpe (MRS) medium containing 15% glycerol at -80°C. A 50-ml Falcon tube containing MRS broth (DIFCO, BD Bioscience, Franklin Lakes, NJ), supplemented with 0.5g/l of cysteine, was inoculated with either a single colony of *B*. *infantis* or *L*. *acidophilus*. The inoculum was cultured at 37°C under anaerobic conditions (BBL Campy Pak Plus Microaerophilic System, Becton, Dickinson, Sparks, MD) until it reached the mid-exponential phase of growth (OD_600_ = 0.5) or the stationary phase of growth (OD_600_ > 1.0), as described previously [26]. Probiotic-conditioned media at those two phases were prepared by centrifugation of probiotic cultures at 9,000 g for 10 minutes, repeated twice, and then passed through 0.22-μm sterile filters to eliminate residual bacteria. The efficiency of bacteria depletion from the conditioned media was determined by plating serial dilutions.

### Cell culture and treatments for microarray analysis

H4 cells, isolated from a 20-week-old normal fetal small intestine, are a human non-transformed primary intestinal epithelial cell line used as an *in vitro* model of the immature intestine [27]. The cells were routinely maintained in DMEM supplemented with 10% heat-inactivated FBS, 1% nonessential amino acid, 1% glutamine, 1% antibiotic/antimycotic solution, 10 mM HEPES buffer, 1 mM sodium pyruvate and 0.2 units/ml human recombinant insulin. Cells were incubated at 37°C in a 5% carbon dioxide, humidified atmosphere. The H4 cells were seeded on a 10-cm-diameter tissue culture-treated dish, cultivated to 90% confluence, then incubated with H4 media containing 15% conditioned media from *B*. *infantis* (BCM) or *L*. *acidophilus* (LCM) for 30 minutes. Without PCM depletion, cells were subsequently incubated with or without IL-1β (10 ng/ml) for 4 hours. The H4 media alone and IL-1β stimulation alone were used as negative and positive controls, respectively. Each experimental condition was completed in triplicate.

### RNA extraction

Following the incubation, H4 cells were lysed in RLT buffer (containing guanidine isothiocyanate) from Qiagen. Total RNA was isolated using an RNeasy kit following the manufacturer’s instructions. By protocol, total RNA was treated with RNase-free DNase I to eliminate genomic DNA contamination. After extraction, RNA quantity was determined by spectrophotometric absorbance of the sample at 260 nm using a NanoDrop spectrophotometer (Thermo Scientific, Wilmington, DE), and purity was determined based on the ratio of absorbance at 260 to that of 280 nm (A_260_/A_280_). Only RNA samples with an A260/280 ratio of 1.8–2.0 were used for further analysis. RNA quality was assessed by subjecting samples to an Agilent 2100 Bioanalyzer (Agilent Technologies, Santa Clara, CA). The bioanalyzer provided a visual inspection of RNA integrity and generated 28S-to-18S ribosomal RNA ratios and an RNA Integrity Number (RIN). An RIN of 10.0 corresponds to a pure, undegraded sample while 1.0 corresponds to a completely degraded sample. Only RNA samples with a RIN score of 6.0 or higher were used for further analysis. Three RNA samples from the following six conditions were evaluated: control, BCM, LCM, BCM/IL-1β, LCM/IL-1β and IL-1β. One sample collected after treatment with BCM/IL-1β was eliminated from processing due to a suboptimal RIN (5.6). The other 17 samples were submitted for standard Affymetrix expression analysis by our core facility of the Harvard Medical School-Partners HealthCare Center for Genetics and Genomics. Samples were stored at -80°C until used for microarray and qRT-PCR.

### Microarray

GeneChip Human Gene 2.0 ST arrays were purchased from Affymetrix (Santa Clara, CA). Preparation of labeled cRNA, hybridization, and scanning of microarray analysis was performed by a core facility, using standard protocols and reagents as described in the Affymetrix Technical Manual (Revision 3).

### Data filtering and analysis

The database was comprised of 17 expression measurements of 53, 618 genes and has been submitted to the Gene Expression Omnibus (accession code: GSE62208). Data were normalized by Robust Multiarray Averaging with a web-based tool (GenePattern, Broad Institute, http://genepattern.broadinstitute.org). The negative control was used as a reference to calculate the fold change of gene expression in the other treatment groups. We defined differentially expressed genes as those whose expression values changed at least twofold (greater than or equal to a 2-fold change) with a p value smaller than or equal to 0.05. These differentially expressed genes were used for further analysis.

A Venn diagram was constructed to overlap the differentially expressed genes that were affected by each treatment. MetaCore (Thomson Reuters, New York, NY) was used to perform gene enrichment analysis by comparing IL-1β with PCM/IL-1β, or PCM with PCM/IL-1β. The top 10 differentially or significantly affected canonical pathways and networks were identified. Fischer’s exact test was used to calculate a p value determining the probability that the association between the genes in the dataset and the canonical pathway was due to chance. Hierarchical clustering analysis was done with a Multi-expression viewer (MEV) software version 4.8 (http://www.tm4.org) on genes that are associated with the nuclear factor-kappa B (NF-κB) activation pathway and extracellular matrix (ECM) remodeling. The fold change expression values were represented in logarithmic scale in a heatmap graph.

### Reverse transcription (RT)

To confirm the microarray findings, the same RNA samples subjected to microarray analysis were used for quantitative RT-PCR. First-strand cDNA synthesis was carried out on 300 ng of total RNA in the final volume of 20 μl with SuperScript III supermix following the manufacturer’s protocol. Briefly, after transfer of 300 ng of total RNA to each nuclease-free microcentrifuge tube, 10 μl of the 2× RT reaction mix, 2 μl of the RT enzyme mix and a variable volume of RNase-free water were added to attain a final volume of 20 μl. The mixture was then incubated at 25°C for 10 minutes, followed by another 30 minutes at 50°C. The reaction was terminated by heating at 85°C for 5 minutes, and then the mixture was chilled on ice. To remove RNA complementary to the cDNA, 1μl of *E*. *coli* RNase H was added and the mixture was incubated at 37°C for 20 minutes. cDNAs were stored at -20°C until use in qPCR.

### Real-time quantitative PCR

Real-time quantitative PCR was carried out on 17 selected genes: IL1B; TNF; RIPK1; BIRC3; IRAK2; TRAF6; NFKBIA; REL; NFKB1; NFKB2; MMP3; MMP12; CD44; PLAU; PLAUR; IL-6 and IL-8, using a StepOnePlus Real-Time PCR System (Applied Biosystem, Carlsbad, CA). Assays were performed with SYBR GreenER qPCR SuperMix Universal following the manufacturer’s instructions. Briefly, in 0.2 ml wells of a 96-well PCR plate, we placed a 25 μl reaction mixture of 12.5 μl SuperMix, 1 μl of forward and reverse primers (10 μM), 1 μl of ROX reference dye, 2 μl of appropriately diluted cDNA and 7.5 μl of sterile distilled water. The thermal cycle conditions consisted of uracil DNA glycosylase activation (50°C, 2 minutes) and DNA polymerase activation (95°C, 10 minutes), followed by 40 cycles of denaturation (95°C, 15 seconds) and then annealing and elongation (60°C, 1minute). Each sample was run in duplicate. A melting curve analysis was used to determine amplification specificity. The primer pairs for the amplification of selected genes were obtained from the Harvard Primer Bank (http://pga.mgh.harvard.edu/primerbank) and Partner’s Genomic DNA core facility at Massachusetts General Hospital. The sequences of the primers are listed in Table 1. Glyceraldehyde-3-phosphate dehydrogenase (GADPH) was used as an endogenous control to normalize for variation in the amount of cDNA template. Results were analyzed using the comparative critical threshold (ΔΔCt) method [28,29] to compare differences between samples. The negative control served as the reference for both microarray and qRT-PCR.

**Table 1:** Primers used in qRT-PCR for quantitation of mRNA expression.

Gene name	Full name	Accession number	5’-3’ sequences
GAPDH	Glyceraldehyde-3-phosphate dehydrogenase	NM_001256799	F: TGTTGCCATCAATGACCCCTT
			R: CTCCACGACGTACTCAGCG
IL1B	Interleukin-1 beta	NM_000576	F: ATGATGGCTTATTACAGTGGCAA
			R: GTCGGAGATTCGTAGCTGGA
TNF	Tumor necrosis factor	NM_000594	F: CCTCTCTCTAATCAGCCCTCTG
			R: GAGGACCTGGGAGTAGATGAG
RIPK1	Receptor (TNFRSF)-interacting serine-threonine kinase 1	NM_003804	F: GGGAAGGTGTCTCTGTGTTTC
			R: CCTCGTTGTGCTCAATGCAG
BIRC3	Baculoviral IAP repeat containing 3	NM_001165	F: TTTCCGTGGCTCTTATTCAAACT
			R: GCACAGTGGTAGGAACTTCTCAT
IRAK2	Interleukin-1 receptor-associated kinase 2	NM_001570	F: CTGCCACCCCAATGTCTTACC
			R: AGGGAACCATTTGCCATGTAG
TRAF6	TNF receptor-associated factor 6	NM_145803	F: TTTGCTCTTATGGATTGTCCCC
			R: CATTGATGCAGCACAGTTGTC
NFKBIA	Nuclear factor of kappa light polypeptide gene enhancer in B-cells inhibitor A	NM_020529	F: CTCCGAGACTTTCGAGGAAATAC
			R: GCCATTGTAGTTGGTAGCCTTCA
REL	V-rel avian reticuloendotheliosis viral oncogene homolog	NM_002908	F: CAACCGAACATACCCTTCTATCC
			R: TCTGCTTCATAGTAGCCGTCT
NFKB1	Nuclear factor of kappa light polypeptide gene enhancer in B-cells 1	NM_003998	F: GAAGCACGAATGACAGAGGC
			R: GCTTGGCGGATTAGCTCTTTT
NFKB2	Nuclear factor of kappa light polypeptide gene enhancer in B-cells 2	NM_001077494	F: AGAGGCTTCCGATTTCGATATGG
			R: GGATAGGTCTTTCGGCCCTTC
MMP3	Matrix metallopeptidase 3	NM_002422	F: CTGGACTCCGACACTCTGGA
			R: CAGGAAAGGTTCTGAAGTGACC
MMP12	Matrix metallopeptidase 12	NM_002426	F: GGAATCCTAGCCCATGCTTTT
			R: CATTACGGCCTTTGGATCACT
CD44	CD44	NM_001001392	F: CTGCCGCTTTGCAGGTGTA
			R: CATTGTGGGCAAGGTGCTATT
PLAU	Plasminogen activator, urokinase	NM_001145031	F: GGGAATGGTCACTTTTACCGAG
			R: GGGCATGGTACGTTTGCTG
PLAUR	Plasminogen activator, urokinase receptor	NM_001005377	F: GAGCTATCGGACTGGCTTGAA
			R: CGGCTTCGGGAATAGGTGAC
IL-6	Interleukin-6	NM_000600	F: TGCAATAACCACCCCTGACC
			R: GCCCAGTGGACAGGTTTCTG
IL-8	Interleukin-8	NM_000584	F: ACCACCGGAAGGAACCATCT
			R: TTTCTGTGTTGGCGCAGTGT

### ELISA assays of IL-6 and IL-8

H4 cells were grown to 90% confluence and treated with 15% BCM, 15% LCM or cell culture medium for 1 hour before being stimulated with IL-1β (1 ng/ml) or exposed to medium. The medium and IL-1β stimulation alone were established as negative and positive controls, respectively. After 18 hours of incubation, the cell culture medium was collected, stored, and subjected to IL-6 and IL-8 measurement by ELISA as described previously [8]. The 96-well high-bond microtitier plates (Nunc-Maxisorp, Fisher Scientific) and human IL-6 and IL-8 detection kits were used for ELISA assays. The data presented are from one of three independent experiments (4 replicates per condition) with similar results.

### Western blot analysis

H4 cells were cultured in 10-cm-diameter dishes. At 90% confluence, cells were incubated with H4 media or media containing 15% BCM or LCM for 30 minutes. Cells were then incubated with or without IL-1β (10 ng/ml) for 1 hour. After stimulation, the cytoplasmic and nuclear proteins were extracted using NE-PER nuclear and cytoplasmic extraction reagents according to the manufacturer’s instructions. Protein concentration was determined by the BCA method using protein assay reagents. Approximately 20 μg of protein was subjected to sodium dodecyl sulfate-polyacrylamide gels electrophoresis and then transferred to polyvinylidene difluoride membranes (Life Technologies, Grand Island, NY). The membranes were blocked in TBST (Tris-buffered saline, 0.1% Tween 20) with 5% nonfat milk for 1 hour at room temperature and then incubated overnight at 4°C with specific primary antibodies such as IκBα (1:500), NF-κB p65 (1:500), β-actin (1:10000) and histone H3 (1:1000). Following washing with TBST three times, membranes were incubated for 1 hour at room temperature with horseradish peroxidase-conjugated secondary antibodies (1:1000). Bound antibodies were detected with ECL reagents. β-actin and histone H3 were used as cytoplasmic and nuclear protein internal controls, respectively. The data presented are from one of three independent experiments (3 replicates per condition) with similar results.

### Immunofluorescence staining of NF-κB p65

H4 cells were seeded on eight-well glass slides (Lab-TekII chamber slide system, Naperville, IL). At 80% confluence, cells were cultured with 15% BCM, 15% LCM or a vehicle for 30 minutes, and then stimulated with 10 ng/mL of IL-1β for 1 hour. The immunofluorescence staining of NF-κB p65 was performed as previously described [24]. Briefly, cells were washed in PBS three times and fixed in 4% paraformaldehyde for 15 minutes. After three washes in PBS, cells were blocked with 5% BSA/PBS for 1 hour and then incubated with the same primary antibody specific for NF-κB p65 (1:200) as used in Western blot analysis. After three washes, cells were incubated with a secondary antibody conjugated with FITC (1:400). Slides were mounted with medium containing DAPI (Invitrogen, Grand Island, NY). Immunofluorescent microscopy was performed via a ×20 Nikon PL APO CS objective mounted on a Nikon Eclipse 80i microscope. The images were captured using NIS-Elements BR 3.2 software (Nikon). This experiment was independently performed three times with 2 replicates in each condition.

### Statistical analysis

For qRT-PCR, ELISA and Western blot analysis, PCM effects were analyzed with one-way ANOVA using SPSS Version 17.0 (SPSS Inc., Chicago, IL) evaluating unstimulated and IL-1β-stimulated conditions separately. When significant differences were noted, a Turkey test was used to compare individual means. The differences between the unstimulated control and IL-1β stimulation alone were determined by the independent t-test. P<0.05 was considered statistically significant.

## Results

### Profiles of gene expression

**Fig 1:**
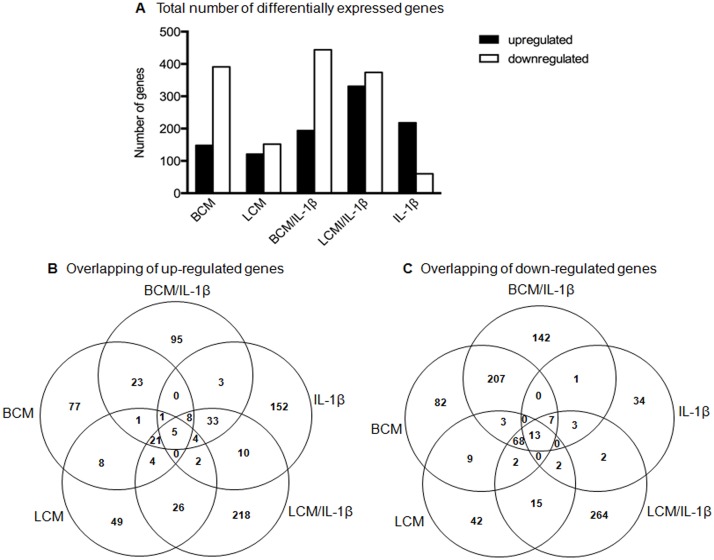
Total number and overlap of differentially expressed genes in H4 cells. The unstimulated control group served as a reference sample to calculate the fold change of each gene after various treatments. Genes with absolute fold-change absolute values over 2.0 were considered upregulated or downregulated. The number of up- and down-regulated genes (A) as well as five-way Venn diagram comparisons of differentially changed genes among various treatment groups (B and C), are shown. Each experimental condition was run in triplicate (n = 3). BCM, *Bifidobacterium infantis*-conditioned media; LCM, *Lactobacillus acidophilus*-conditioned media.

The transcriptional profile of genes that were either up- or down-regulated at least twofold with each treatment compared to the negative control is shown in Fig 1. Despite IL-1β stimulation, BCM treatment led to a larger number of downregulated genes than upregulated genes (Fig 1A). In contrast, the IL-1β stimulation alone induced 218 upregulated genes, which was more than triple the number of downregulated genes (60). The numbers of up- and down-regulated genes were similar in both LCM and LCM/ IL-1β treatments. Two five-way Venn diagrams were constructed to further examine the profiles of gene expression by overlapping the up- and down-regulated genes (Fig 1B and 1C). Overlapping areas represent genes that are modified by more than one condition. There were only 5 out of 740 (0.68%) upregulated and 13 out of 896 (1.45%) downregulated genes common among all conditions, suggesting that condition-specific changes in gene expression exist. Of the 283 upregulated genes in BCM-treated cells, 20.85% (59 out of 283) remained upregulated despite IL-1β stimulation. Similarly, 15.90% (62 out of 390) of the upregulated genes in LCM-treated cells persisted despite IL-1β stimulation. The Venn diagram in Fig 1B showed that 12.73% of the upregulated genes in both BCM/IL-1β and BCM conditions overlapped and 15.08% were upregulated in both LCM/IL-1β and LCM conditions. Regardless of IL-1β stimulation, BCM strongly downregulated gene expression, reflected by 298 out of 537 (55.49%) common genes between BCM and BCM/IL-1β groups. IL-1β stimulation alone shared the fewest downregulated genes with other treatments.

### Pathway analysis of differentially expressed genes

**Fig 2:**
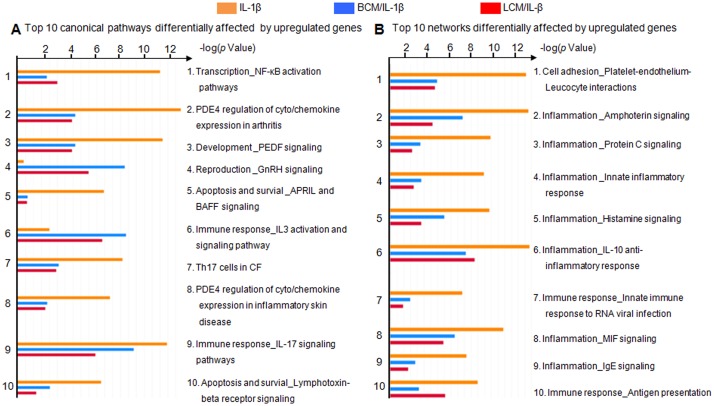
GeneGo MetaCore gene enrichment analysis of upregulated genes in H4 cells. The top 10 pathways (A) and networks (B) differentially affected between the negative control and probiotic-conditioned media treatments under IL-1β stimulation were obtained from the analysis. BCM, *Bifidobacterium infantis*-conditioned media; LCM, *Lactobacillus acidophilus*-conditioned media. NF-kB, nuclear factor-kappa B; PDE4, cAMP-specific phosphodiesterase-4 family protein group; PEDF, pigment epithelium-derived factor protein; GnRH, gonadotropin-releasing hormone; APRIL, tumor necrosis factor (ligand) superfamily members 13; BAFF, tumor necrosis factor (ligand) superfamily members 13b; CF, cystic fibrosis; MIF, macrophage migration inhibitory factor.

A MetaCore gene enrichment analysis based on the significantly affected genes was performed. Ranking of relevant integrated pathways was based on hypergeometric p values. Lower p values are associated with increased relevance of the pathways. In Fig 2, the top 10 differentially affected pathways and networks in IL-1β-stimulated cells with and without PCM pretreatment are listed. PCM treatment alone was absent from this analysis as it showed a low proportion of upregulated genes common with the IL-1β-stimulated groups (as shown in Fig 1B). Three of the top 10 pathways are associated with the immune response, three with autoimmune or lung diseases, two with apoptosis and cell survival, and one with reproduction. Pigment epithelium-derived factor signaling is involved in multiple cellular processes including apoptosis and cell survival, cell cycle, development, angiogenesis and immune response. Among all top pathways, the canonical and non-canonical NF-κB activation pathways were the most differentially affected. The p values of eight pathways affected by PCM pretreatments were higher compared to IL-1β stimulation alone, with the exception of gonadotropin-releasing hormone signaling and IL-3 activation and signaling pathways (Fig 2A). The top 10 networks differentially affected were associated with cell adhesion and inflammation. The p values of the top 10 networks in IL-1β-stimulated cells pretreated with PCM were all higher than IL-1β stimulation alone (Fig 2B), indicating less involvement of these networks.

**Fig 3:**
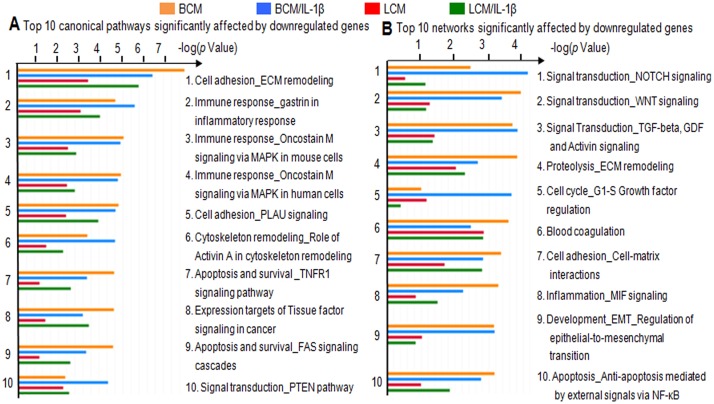
GeneGo MetaCore gene enrichment analysis of down-regulated genes in H4 cells. The top 10 pathways (A) and networks (B) significantly affected by probiotic-conditioned media treatments with and without IL-1β stimulation are listed. BCM, *Bifidobacterium infantis*-conditioned media; LCM, *Lactobacillus acidophilus*-conditioned media. ECM, extracellular matrix; MAPK, mitogen-activated protein kinase; PLAU, urokinase-type plasminogen activator protein; TNFR, TNF-α receptor; FAS, Fas cell surface death receptor; PTEN, phosphatase and tensin homolog, TGF-beta, transforming growth factor-beta; GDF, growth differentiation factor; MIF, macrophage migration inhibitory factor; EMT, epithelial-mesenchymal transition; NF-kB, nuclear factor-kappa B.

As shown in Fig 1C, the IL-1β-stimulated group shared very few downregulated genes with the other four groups, whereas the PCM treatment with or without IL-1β stimulation shared a high ratio of downregulated genes. Therefore, the pathway analysis of downregulated genes in PCM and PCM/IL-1β groups was performed and IL-1β stimulation alone was excluded from this analysis. The top 10 significantly affected pathways and networks are shown in Fig 3. Seven of the top 10 pathways were associated with cell adhesion, immune response, and apoptosis and cell survival. Two of them were related to protein function and one to neoplastic processes. The ECM remodeling pathway was the most significantly downregulated, demonstrated by the lowest p value. In addition, regardless of IL-1β stimulation, BCM treatment had slightly lower p values than LCM treatment, with the exception of the PTEN pathway (Fig 3A). In terms of networks, the top three downregulated networks were those associated with signal transduction with NOTCH, WNT and TGF-β, GDF and activin signaling. Other networks were relevant to cell adhesion, cell cycle, apoptosis and inflammation. Similarly, the lower p values of BCM compared to LCM treatment possibly suggest a stronger gene expression modulatory effect by BCM (Fig 3B).

### Genes associated with NF-κB activation pathways and ECM remodeling

**Fig 4:**
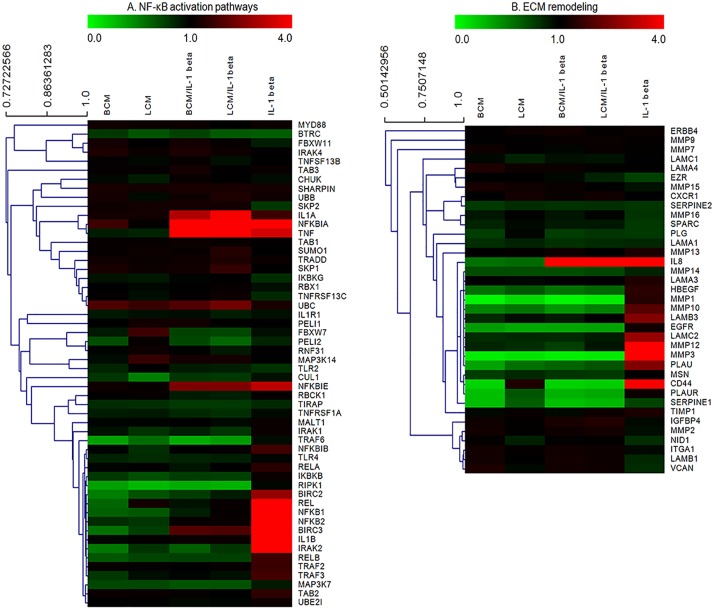
Hierarchical clustering of genes involved in NF-κB activation (A) and ECM remodeling (B). Expression levels of each gene in the various treatment groups relative to the unstimulated control group are displayed as fold changes. Red or green color indicates the fold change of up- or down-regulated expression levels, respectively, compared with unchanged expression shown in black. BCM, *Bifidobacterium infantis*-conditioned media; LCM, *Lactobacillus acidophilus*-conditioned media.

Subsequently, we chose to further analyze the most differentially upregulated (NF-κB activation) and significantly downregulated (ECM remodeling) pathways, using hierarchical clustering of genes. The PCM-induced changes of gene expression within these two pathways are shown in Fig 4. In both pathways, hierarchical clustering of organized genes fell into three groups: downregulated genes seen in PCM treatment alone; upregulated genes seen in IL-1β stimulation alone; both up- and down-regulated genes seen in IL-1β-stimulated cells pretreated with PCM. In terms of NF-κB activation pathways (Fig 4A), IL-1β stimulation alone significantly upregulated the expression of IL1B, BIRC3, IRAK2, REL, NFKB1 and NFKB2 (fold change ≥ 2), while PCM treatment resulted in either no change (fold change < 2) or downregulation of the levels of these genes. Additionally, PCM treatment significantly induced NFKBIA expression, and suppressed RIPK1 and TRAF6 expression compared with IL-1β stimulation alone. Fig 4B shows that the gene expression of the following genes is differentially modulated by PCM treatment compared with IL-1β stimulation alone: MMP14, HBEGF, MMP1, MMP10, LAMB3, EGFR, LAMC2, MMP12 MMP3, PLAU, CD44, PLAUR and SERPINE1.

### Validation of differentially expressed target genes by qRT-PCR

**Fig 5:**
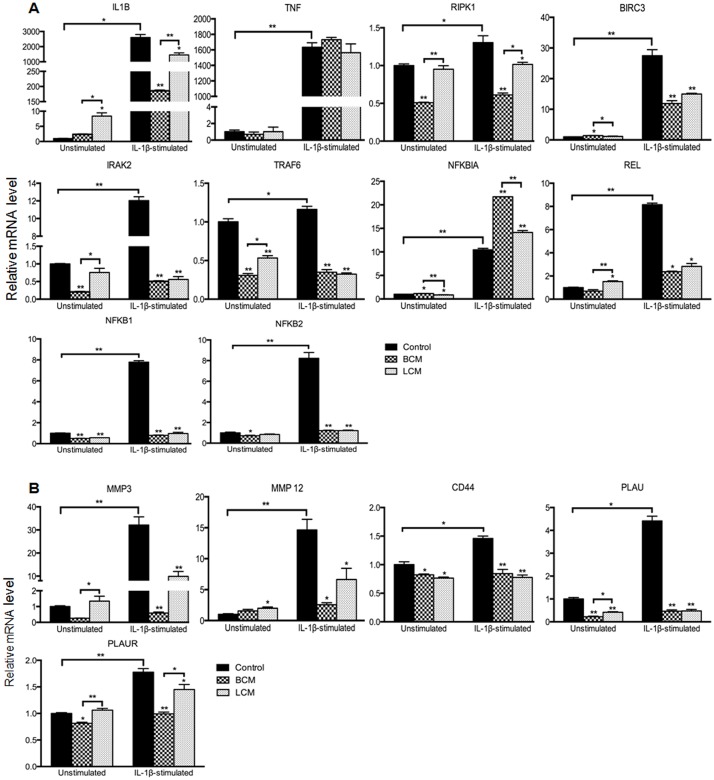
Validation of microarray results of selected genes in H4 cells by qRT-PCR. The RNA used for qRT-PCR was from the same samples used for microarray analysis. NF-κB activation (A) and ECM remodeling (B) associated genes with significant changes are shown. Expression levels of genes are normalized to glyceraldehyde-3-phosphate dehydrogenase (GADPH). Relative mRNA levels were calculated using the 2^-ΔΔCt^ method and the average ΔCt values of the unstimulated control group served as the calibrator. The gene expression levels of probiotic-conditioned media treatments were compared to the corresponding control group, unstimulated and IL-1β-stimulated, respectively. The data represent the mean ± the SEM (n = 3). A p<0.05 (*) or p<0.001 (**) depicts the significance value. BCM, *Bifidobacterium infantis*-conditioned media; LCM, *Lactobacillus acidophilus*-conditioned media.

Fifteen target genes involved in NF-κB activation and ECM remodeling were selected on the basis of their magnitude of change and differences among treatments. These genes were evaluated by qRT-PCR to confirm the transcription profiling results. Differential gene expression was consistent between the two independent methods of analysis and the expression of the 15 genes changed in the same direction, although there were quantitative differences between the gene array analysis and qRT-PCR results (Fig 5). For example, compared with IL-1β stimulation alone, TNF expression was not decreased whereas IRAK2 expression was reduced by PCM/IL-1β treatments, using both analytic methods. The expression of NFKBIA, which encodes IκBα, was induced by PCM treatments in IL-1β stimulated cells. These findings confirmed our microarray data, demonstrating that PCM treatment modulated the gene expression patterns associated with NF-κB activation and ECM remodeling during IL-1β stimulation.

### The mRNA and protein expression levels of IL-6 and IL-8

**Fig 6:**
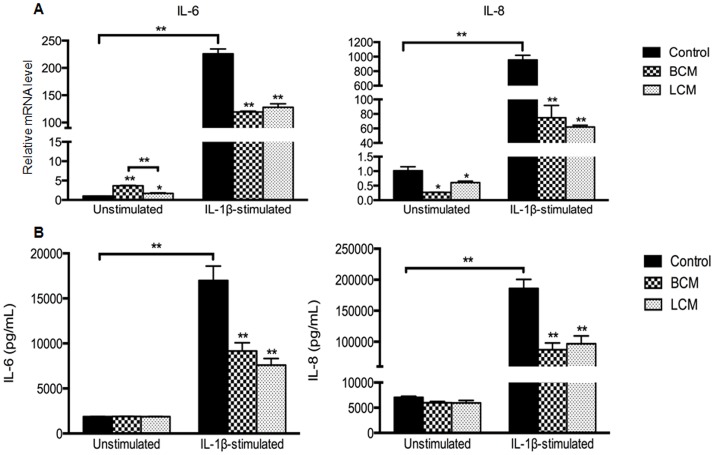
The mRNA and protein expression of IL-6 and IL-8 in H4 cells. Gene expression was determined by qRT-PCR (A) and protein level was measured by ELISA (B). Expression levels of genes are normalized to glyceraldehyde-3-phosphate dehydrogenase (GADPH). Relative mRNA levels were calculated using the 2^-ΔΔCt^ method and the average ΔCt values of the unstimulated control group served as the calibrator. The gene expression and cytokine production in probiotic-conditioned media treatments was compared to the corresponding control group, unstimulated and IL-1β-stimulated, respectively. All data represent the mean ± the SEM (n = 3 for qRT-PCR and n = 4 for ELISA). A p<0.05 (*) or p<0.001 (**) depicts the significance value. BCM, *Bifidobacterium infantis*-conditioned media; LCM, *Lactobacillus acidophilus*-conditioned media.

Consistent with the IL-6 and IL-8 mRNA expression determined by qRT-PCR, the cellular secretion of IL-6 and IL-8 in IL-1β-stimulated cells measured by ELISA was significantly decreased by both BCM and LCM treatments (p<0.05) (Fig 6). This was also consistent with the transcription profiling results.

### Degradation of cytoplasmic IκBα and nuclear translocation of NF-κB p65

**Fig 7:**
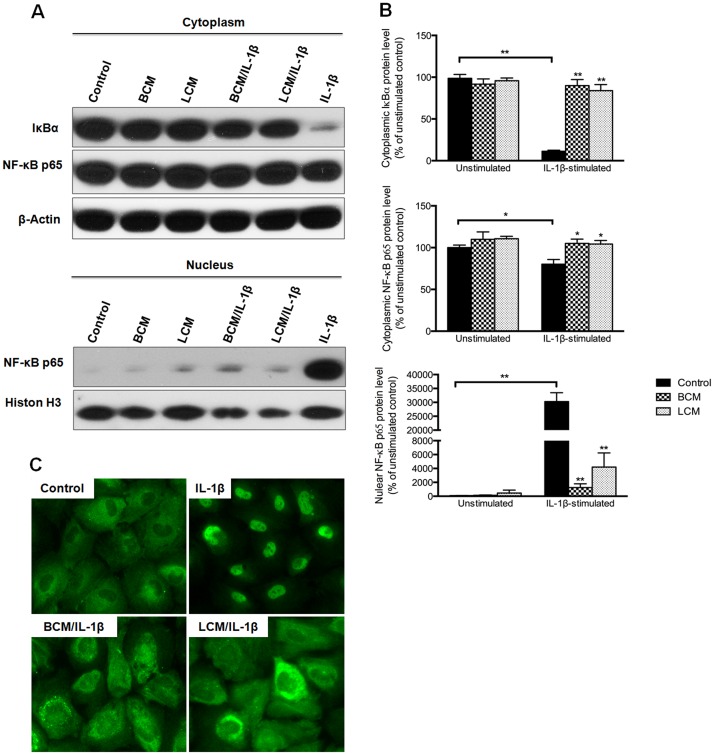
Degradation of cytoplasmic IκBα and nuclear translocation of NF-κB p65 in H4 cells. Western blot was performed and densitometry of immune blot bands was used for quantification. The protein levels of cytoplasmic IκBα, as well as cytoplasmic and nuclear NF-κBp 65 (A) and quantification of each immuno blot band (B) are displayed. The protein levels in probiotic-conditioned media treatments were compared to the corresponding control group, unstimulated and IL-1β-stimulated, respectively. All data represent the mean ± the SEM (n = 3). A p<0.05 (*) or p<0.001 (**) depicts the significance value. Immunofluorescence staining of NF-κB p65 (green) was performed in H4 cells (C). The fields presented were randomly captured to accurately represent each condition. BCM, *Bifidobacterium infantis*-conditioned media; LCM, *Lactobacillus acidophilus*-conditioned media.

Western blot analysis showed that IL-1β stimulation significantly decreased cytoplasmic IκBα and NF-κB p65 levels, and increased nuclear NF-κB p65 expression compared with the unstimualted control (p<0.05) (Fig 7A and 7B). However, both BCM and LCM treatments significantly inhibited the degradation of IκBα and nuclear translocation of NF-κB p65 induced by IL-1β stimulation (p<0.05). To directly observe the translocation of NF-κB p65, immunofluorescence staining was performed and the images are shown in Fig 7C. IL-1β stimulation of H4 cells induced a dramatic translocation of NF-κB p65 from the cytoplasm to the nucleus, compared to the unstimulated control. BCM and LCM treatments successfully prevented the majority of NF-κB p65 translocation in IL-1β-stimulated cells.

## Discussion

It is understood that the intestine of premature infants is functionally immature. We have previously demonstrated that the innate immune profile of pro-inflammatory and anti-inflammatory mediators within the immature intestine is inappropriately skewed toward a pro-inflammatory state [9]. When a newborn infant is exposed to microbes during the process of intestinal bacterial colonization, the immaturity of the intestinal inflammatory profile matures and the intestine adopts appropriate responsiveness. Accordingly, intestinal immunity is required to accept colonizing bacteria and nutritional antigens, while rejecting potential pathogens. This physiologic process occurs relatively rapidly in the newborn infant. However those born prematurely are more likely to mount erratic inflammatory responses to even commensal bacteria. It is felt that this erratic responsiveness may contribute to the devastating intestinal inflammation seen in NEC [30]. Additionally, the pathophysiology of NEC is multifactorial and primarily consists of genetic predisposition, intestinal immaturity, and an imbalance in microvascular tone, accompanied by an increased risk of virulent colonizing bacteria and an exaggerated inflammatory response to commensal intestinal bacteria [1,3].

Given the complex pathophysiology of NEC, it is difficult to determine the exact mechanisms by which probiotic bacteria reduce its incidence, as shown in clinical trials. Certainly, there is mounting evidence that these beneficial microbes modulate intestinal inflammatory responses. In poly (I·C) challenged human intestinal epithelial cells (HT-29 cells), a multistrain probiotic regimen of *L*. *helveticus* R0052, *B*. *longum* subsp. *infantis* R0033 and *B*. *bifidum* R0071 attenuated the expression of genes associated with proinflammatory T_H_1 and antiviral innate immune responses [31]. The primary modulated pathways included the TLR3 domain-containing adapter-inducing beta interferon, mitogen-activated protein kinase and NF-κB signaling pathways. In another transcriptional study of immune signaling pathways in HT-29 cells, seven probiotic strains of *Bifidobacterium* spp. and *Lactobacillus* spp. exerted anti-inflammatory effects by differentially regulating the central mitogen-activated protein kinase signaling pathways [32]. A whole genome microarray experiment showed the beneficial effects of *L*. *rhamnosus* GG and *L*. *acidophilus* on gut responses in a neonatal gnotobiotic pig model. Immune function-related genes were down- and up-regulated in the early (day 1) and late (day 7) stages of colonization, respectively [33]. Using cDNA microarray analysis from the intestine of 2-week-old mice orally inoculated with live *Lactobacillus rhamnosus* GG (LGG), Lin et al. showed an up-regulated battery of antiapoptotic and cytoprotective genes [17].

Conditioned media from *L*. *acidophilus*, *B*. *infantis* and *L*. *plantarum* were also shown to have anti-inflammatory and cytoprotective properties on intestinal cells, in both *in vivo* and *in vitro* studies conducted previously [34–37]. The conditioned medium from co-cultured *B*. *infantis* and *L*. *acidophilus* was previously demonstrated to be anti-inflammatory using *in vitro* models of NEC and the functional component of this medium was a 5–10 kDa glycolipid or glycan, resistant to DNase, RNase, protease, heat stress and acid exposure [8]. However, the pathways mediating this anti-inflammatory effect remain unclear. In addition, our more recent work indicated distinct differences in the ability of BCM and LCM to attenuate inflammation using the same *in vitro* models, suggesting strain specific modulation of inflammatory pathways. Therefore, in this *in vitro* study, we reported the use of microarray analysis to investigate the effects of BCM and LCM on gene expression in IL-1β-stimulated immature human enterocytes (H4 cells).

Genome-wide expression profiles of human NEC tissues showed significant and extensive changes of gene expression, particularly from pathways associated with angiogenesis, cell adhesion and chemotaxis, extracellular matrix remodeling, inflammation and muscle contraction [38]. This was consistent with our results using a human immature enterocyte model of NEC. Although most of the pathways and networks affected by PCM in H4 cells, shown in Fig 2, involved immune response and inflammation, others belonged to several cellular processes including autoimmune or lung diseases, apoptosis and survival, reproduction, cell cycle, development and angiogenesis.

Despite IL-1β stimulation, PCM treatments were shown to be potent downregulators of several genes associated with cell adhesion, immune response, apoptosis and cell survival, and cell cycle (Fig 3). Downregulation of extracellular matrix remodeling of cell adhesion and cell cycle may inhibit cell death. This is consistent with the observation by Yanagihara et al. showing that short exposure (4 hours) of Caco-2 cells to *L*. *acidophilus* L-92 repressed genes involved in cell growth and cell meiosis [39]. Using microarray DNA chips, Tien et al. also observed that *L*. *casei* downregulated the transcription of several genes encoding pro-inflammatory cytokines and adherence molecules induced by invasive *Shigella flexneri* [40]. Additionally, Caco-2 cells incubated with *L*. *casei* for 24 hours significantly affected the expression of genes involved in cell cycle, apoptosis, hypoxia and ubiquitination/degradation pathways. Using an *in vivo* model of immature intestinal inflammation, conditioned media from *B*. *infantis* was reported to decrease the percentage of apoptotic cells in *C*. *sakazakii*-challenged newborn mice by reducing caspase 3 and 7 activation [24]. Consistently, the present microarray data of H4 cells showed that expression of caspase 3 was downregulated more than two fold by both BCM and LCM during IL-1β stimulation (p<0.05).

The extracellular matrix (ECM) plays a critical role in organogenesis and tissue remodeling by serving as a structural support and a medium for cell-cell interactions. ECM remodeling is associated with morphogenetic events during embryonic development and regeneration [41–43]. Matrix metalloproteases (MMPs) that degrade ECM components are a superfamily of matrix-degrading proteases, including collagenases (MMP1 and MMP13), gelatinases (MMP2 and MMP9), stromelysins (MMP3 and MMP10), matrilysins (MMP7), membrane type MMPs (MMP14, MMP15 and MMP16) and other MMPs (MMP12) [44]. The expression levels of MMP1, 3, 10, and 14 were unchanged or upregulated in IL-1β stimulated H4 cells, but were downregulated in PCM-treated H4 cells, with or without IL-1β stimulation.

Several reports observed that the use of probiotics reduced the expression of MMP2 and MMP9, which played an important role in the inflammatory process and associated disease [45–47]. Other than MMPs, tissue-type plasminogen activator urokinase (PLAU) also participates in cell migration and tissue remodeling. It binds to its receptor (PLAUR) and mediates a variety of functions involved in vascular homeostasis, inflammation and tissue repair [48]. Both PLAU and PLAUR were downregulated by PCM treatment, while upregulated or unchanged by IL-1β stimulation. There are no previous observations reported in the literature regarding the effects of probiotic administration on PLAU and PLAUR expression.

NF-κB signaling in intestinal epithelial cells plays a critical role in the maintenance of immune homeostasis by regulating cell survival, barrier integrity, as well as immunological and anti-microbial responses [49]. Pathways of NF-κB activation in response to different stimuli are broadly classified as canonical and non-canonical pathways, depending on whether activation involves I-κB degradation or NF-κB2 (p100) processing. IL-1β is one of the most potent stimuli of the NF-κB canonical pathway. In HT-29 human epithelial cells and *in vivo* murine investigations, *B*. *infantis* was demonstrated to exert protection against flagellin or pathogen stimulation by inhibiting NF-κB activation [50,51]. Culture supernatant of *L*. *acidophilus* decreased the nuclear p65 NF-κB levels by attenuating Bcl10 interaction with MALT1 and ubiquitination of IKKγ in platelet-activating factor-treated intestinal epithelial cells [52]. Additionally, in *Helicobacter pylori* infected MKN45 cells, pretreatment with *L*. *acidophilus* reduced IL-8 expression by increasing cytoplasmic IκBα and decreasing nuclear translocation of NF-κB [53].

We have demonstrated that NFKB1 and NFKB2 expression, significantly upregulated by IL-1β stimulation, was downregulated with PCM treatment prior to stimulation. Additionally, the attenuation of NF-κB responses by PCM treatment included downregulation of several molecules (IL1B, BIRC2, BIRC3, IRAK2, TRAF2, TRAF3, TRAF6, RIPK1 and REL), as well as upregulation of NFKNIA, which encoded IκBα. IκBα binds NF-κB and prohibits either the nuclear translocation or the DNA binding of the transcription factor [54]. The high expression of IκBα induced by PCM treatment could be useful in blocking NF-κB signaling pathways. Given the developmental regulation of IκB expression, we elected to further investigate the maturational effects of BCM and LCM on this particular pathway.

A confirmatory Western blot analysis of IκBα and NF-κB p65 was included in our investigation in order to demonstrate an associated post-transcriptional modification. As expected, IL-1β stimulation caused a dramatic increase of IκBα degradation and nuclear NF-κB p65 levels. This is consistent with prior studies showing a significant increase in NF-κB activity in nuclear extracts from 30 minutes to 4 hours in IL-1β-stimulated Caco-2 cells [55]. In our study, PCM treatment attenuated the nuclear translocation of NF-κB p65 by maintaining the cytoplasmic IκBα level in stimulated H4 cells. The conditioned media from *B*. *infantis* used in the current study was also demonstrated to maintain IκBα levels in the ileum of *C*. *sakasakii*-infected newborn mice and decrease *C*. *sakasakii*-triggered nuclear translocation of NF-κB p65 in H4 cells [24].

In conclusion, conditioned media from *B*. *infantis* or *L*. *acidophilus* protected the immature human fetal intestinal epithelial H4 cells against IL-1β stimulation by affecting several cellular processes. The decreased expression of ECM remodeling-associated genes, mainly MMPs, likely contributes to stabilization of cells and reduction of inflammation. The anti-inflammatory effects of PCM were primarily mediated by attenuating NF-κB activation. It is particularly intriguing to demonstrate a beneficial modulation of the NF-κB pathway, as immaturities of this pathway have been of particular interest in the pathogenesis of NEC. Further studies will be designed to investigate the anti-inflammatory effects of BCM and LCM using gene specific *in vivo* knockout NEC models. Continued understanding of these effects will allow an educated decision regarding the applicability of PCM as a potential preventive strategy against NEC.
